# Essential components of postnatal care – a systematic literature review and development of signal functions to guide monitoring and evaluation

**DOI:** 10.1186/s12884-022-04752-6

**Published:** 2022-05-28

**Authors:** Hannah McCauley, Kirsty Lowe, Nicholas Furtado, Viviana Mangiaterra, Nynke van den Broek

**Affiliations:** 1 Liverpool School of Tropical Medicine, Pembroke Place, L3 5QA UK; 2 The Global Fund for Aids Tuberculosis and Malaria, Switzerland Geneva,; 3 Bocconi School of Management, Bocconi University, Milan, Italy

**Keywords:** Postnatal care, Maternal morbidity, Neonatal morbidity, Global health, Health services, Quality of care

## Abstract

**Background:**

Postnatal Care (PNC) is one of the healthcare-packages in the continuum of care for mothers and children that needs to be in place to reduce global maternal and perinatal mortality and morbidity. We sought to identify the essential components of PNC and develop signal functions to reflect these which can be used for the monitoring and evaluation of availability and quality of PNC.

**Methods:**

Systematic review of the literature using MESH headings for databases (Cinahl, Cochrane, Global Health, Medline, PubMed, and Web of Science). Papers and reports on content of PNC published from 2000–2020 were included. Narrative synthesis of data and development of signal function through 7 consensus-building workshops with 184 stakeholders.

**Results:**

Forty-Eight papers and reports are included in the systematic review from which 22 essential components of PNC were extracted and used to develop 14 signal functions. Signal functions are used in obstetrics to denote a list of interventions that address major causes of maternal and perinatal morbidity or mortality. For each signal function we identified the equipment, medication and consumables required for implementation. The prevention and management of infectious diseases (malaria, HIV, tuberculosis) are considered essential components of routine PNC depending on population disease burden or whether the population is considered at risk. Screening and management of pre-eclampsia, maternal anaemia and mental health are recommended universally. Promotion of and support of exclusive breastfeeding and uptake of a modern contraceptive method are also considered essential components of PNC. For the new-born baby, cord care, monitoring of growth and development, screening for congenital disease and commencing vaccinations are considered essential signal functions. Screening for gender-based violence (GBV) including intimate partner- violence (IPV) is recommended when counselling can be provided and/or a referral pathway is in place. Debriefing following birth (complicated or un-complicated) was agreed through consensus-building as an important component of PNC.

**Conclusions:**

Signal functions were developed which can be used for monitoring and evaluation of content and quality of PNC. Country adaptation and validation is recommended and further work is needed to examine if the proposed signal functions can serve as a useful monitoring and evaluation tool.

**Trial registration:**

The systematic review protocol was registered: PROSPERO 2018 CRD42018107054.

**Supplementary Information:**

The online version contains supplementary material available at 10.1186/s12884-022-04752-6.

## Background

Postnatal Care (PNC) is one of the care packages that make up the continuum of care for mothers and babies globally [1, 2]. A significant number of maternal deaths still occur during the postnatal period and an estimated 2.8 million babies die in the first month of life (neonatal death) [3, 4]. Neonatal deaths account for up to 52% of all deaths in children under-5 years of age [5]. The majority of maternal and neonatal deaths are treatable and preventable with timely recognition and good-quality care [6].

Current guidelines advise that women should have at least eight ANC visits or contacts during pregnancy, a skilled attendant with adequate resources at the time of birth, and PNC immediately after birth and/or on at least four occasions in the subsequent six weeks [2, 7, 8].

Despite the critical importance of the postnatal period for both maternal and child survival and well-being, PNC consistently has the lowest coverage rates [9]. Postnatal care coverage is not a reported in the annual World Health Statistics reports and nor is it a component of the indicator to assess Universal Health Coverage (UHC). Estimates show that globally far fewer women and newborn babies receive PNC compared to antenatal care (ANC), with less than half of women receiving a postnatal care visit within two days of childbirth [10, 11].

It is recommended that women who give birth with a skilled attendant in a healthcare facility receive immediate postnatal care and stay at the healthcare facility for at least 24 hours in case of uncomplicated birth [12]. However, it has been reported that even when women give birth in a healthcare facility, this may not include PNC as women may only stay at the healthcare facility for a few hours [13]. Of the 48% of women in sub-Saharan Africa who give birth without a skilled birth attendant only 13% receive a PNC visit [14].

The importance of PNC for reducing neonatal mortality has been documented with an estimation that if PNC rates were to reach 90% in sub-Saharan Africa, then 10–27% of all neonatal deaths could be averted [15]. Research has similarly outlined the considerable extent of maternal psychological and physiological morbidity following childbirth especially among vulnerable populations [16, 17]. These include maternal anaemia, hypertension, puerperal and other infections as well as the need for increased psychosocial support. Timely identification and management during and after pregnancy can reduce the burden of disease and prevent complications particularly where morbidity and mortality levels among women of reproductive age are high [18].

In addition to the screening, identification, and management of pregnancy- and birth-related morbidity, the postnatal period and postnatal care package is an opportunity for the promotion and implementation of other components of public health, including the commencing of childhood immunisations, exclusive breastfeeding and uptake of modern contraceptive methods [1]. Care in the first 1000 days of life is crucially important to ensure that children survive and thrive. Children who are exclusively breastfed are 14 times more likely to survive the first six months of life than non-breastfed children [19]. Receiving PNC is significantly associated with modern contraception use [2, 20].

PNC is also an important platform for programmes that aim to tackle the inequities in HIV, tuberculosis and malaria prevention and treatment [21]. For example, mother-to-child transmission of HIV (PMTCT) programmes provide treatment and education to HIV positive mothers and treatment for HIV-exposed infants with the aim of preventing newborn infections [22]. In high-burden settings, nearly half of all new HIV infections among children occur during the postnatal period. However, this is also when many women who are HIV positive fail to attend for ongoing care and treatment and drop out of such programmes. This means that comparatively more infant HIV infections occur during the postnatal period than during pregnancy and labour [23].

It is important that all components of PNC are provided to the mother and her baby in an integrated holistic manner. Given the low coverage rates and uptake of PNC globally, the attention internationally has been largely on supporting the implementation and uptake of at least the minimum number of PNC visits that are considered effective, and, on where and who can provide PNC at the healthcare facility level as well as in the community [24]. There has been less emphasis on the essential components or minimum content of the PNC care package required to meet the needs of both mothers and/or babies. Without the right content PNC will largely remain a ‘missed opportunity’. For other care packages that make up the continuum of care including for Emergency Obstetric Care and Antenatal Care ‘signal functions’ have been developed which reflect the essential components of a care package [25, 26]. These have however not yet been developed for PNC.

We therefore conducted a systematic review of the literature and consensus-building workshops with a range of key stakeholders to identify the essential components of PNC and develop signal functions to assist in the monitoring and evaluation of availability and quality of PNC.

## Methods

The PRISMA guidelines were followed for this systematic review and a narrative summary of results is provided [27]. The World Health Organisation (WHO) definition of postnatal period is ‘postnatal period begins immediately after the birth of the baby and extends up to six weeks (42 days) after birth’. When describing care provision, the postnatal period consists of immediate, early and late periods. The period from days 2 through 7 is defined as the early postnatal period and the period from days 8 through 42 as the late postnatal period [2].

The review protocol was registered (PROSPERO 2018 CRD42018107054).

### Search strategy

A systematic search strategy was developed. Six databases including Cinahl, Cochrane (Cochrane Database of Systematic Reviews and the Cochrane Central Register of Controlled Trials), Global Health, Medline, PubMed, and Web of Science were searched using MeSH Headings, subheadings, thesaurus, and key word searches. A librarian was involved in developing MeSH terms and selecting relevant databases. Bibliographies from the articles selected for full text retrieval were reviewed to identify additional relevant studies. Key word searches were also conducted in Google Scholar. (Table S1 - Search Terms).

### Inclusion criteria

Articles from indexed journals describing one or more components of the content of PNC were included. As the researchers were English and French speaking, articles were limited to those published in English and French published between January 2000 (to coincide with the development of the postnatal care guidance from WHO) and September 2020.

### Exclusion criteria

Studies that did not describe at least one content component of PNC were excluded. Studies were also excluded if they were case studies or research protocols. Finally, studies with no research methods such as journalistic style articles, editorials and individual volunteer accounts including personal reflection accounts were excluded.

### Terminology

For this review, we used the term ‘component’ to denote individual interventions or actions that are considered part of the PNC care package. The terms ‘core’, ‘key’, ‘vital’ and ‘essential’ are used interchangeably in the literature. For the purpose of this review, we use the term ‘essential’ to denote a requirement for the PNC care package. In obstetrics ‘signal functions’ are used to denote a representative shortlist of key interventions and activities that address major causes of maternal and perinatal morbidity or mortality [25, 26]. These were first used in obstetrics to define Emergency Obstetric Care with nine identified signal functions describing this care package. A list of signal functions does not include every service that may need to be provided but are considered as representative of a minimum essential care package that needs to be in place. The equipment, medication and/or vaccines required to implement each signal function can be identified and must be in place to be able to provide each relevant component of care.

Both the words ‘postpartum’ and ‘postnatal’ are used in the literature and in policy documents sometimes interchangeably. The WHO recommends the adoption of just a single term ‘postnatal’ to be used for all issues pertaining to the mother and the baby after birth up to 6 weeks (42 days) [28]. A distinction is made between ‘immediate postnatal care’ which is given immediately after birth and in the first 24-h after birth before discharge home (if birth is in a healthcare facility). Subsequent PNC is also referred to as ‘routine’ PNC visits and is recommended on at least three further occasions; day-3, days 7–14 and 6-weeks after birth [12]. This systematic review and developed signal functions pertain to routine PNC visits.

### Study selection

Papers identified from the electronic searches were imported into Endnote and duplicates were removed. Three independent researchers reviewed all titles and abstracts to determine if papers met the inclusion criteria. Where inclusion/exclusion criteria could not be determined from the titles and abstracts alone and/or for papers without an abstract, full articles were retrieved and reviewed for relevance. In case of uncertainty or discordance between reviewers the full text was reviewed again by all three and consensus reached to include or exclude.

Quality assessment was undertaken on all included papers using The Hawker et al. Critical Appraisal Tool. The checklist is used to assess nine areas of the research article. The maximum score an article can score is 36 for fulfilling all the items on the checklist while a minimum score of 9 can be scored for a very poor article.

### Data extraction

Using a pre-designed data extraction form, information for each study was extracted by two independent researchers to include type of study, population, and the individual components of PNC recommended or assessed. Any disagreement was resolved by discussion with a third researcher. Information obtained from studies that reported on more than one component of PNC was recorded in a central summary table. Studies that reported on only one component of PNC were summarised in separate tables by themes which were identified during review.

### Data synthesis

A narrative synthesis was used to summarise findings. All identified individual components of PNC were listed and were categorised to develop a draft list of 25 signal functions with identification of the equipment, medication and consumables required to deliver each. A series of consensus-building workshops were held (3 international and 4 national) with a range of stakeholders (184 in total) including researchers, clinicians, health service managers from low- and middle-income countries (Afghanistan, Chad, Ghana, Togo, Nigeria) as well as high income settings (Europe, USA, UK) and key representatives from UN partners (UNFPA, WHO, UNICEF, and the Global Fund). Workshops were organised to allow for examination of each recommendation for content of PNC in small working groups followed by plenary discussion, consensus agreement leading to adoption or not of proposed content, signal function, equipment, medication and consumables required for each. Consensus-building workshops were conducted alongside and during the ongoing systematic review with evidence obtained from review of documents (policy, guidelines) and peer reviewed papers presented at time of the workshops. Adaptation or not of any component as discussed during any workshop was based on evidence where available with practices for which there was evidence of non-effectiveness or harmful practices agreed as needing to be discarded. After synthesis of all workshop feedback and the literature review a comprehensive list of 15 proposed signal functions were developed.

## Results

### Description of studies

**Fig. 1:**
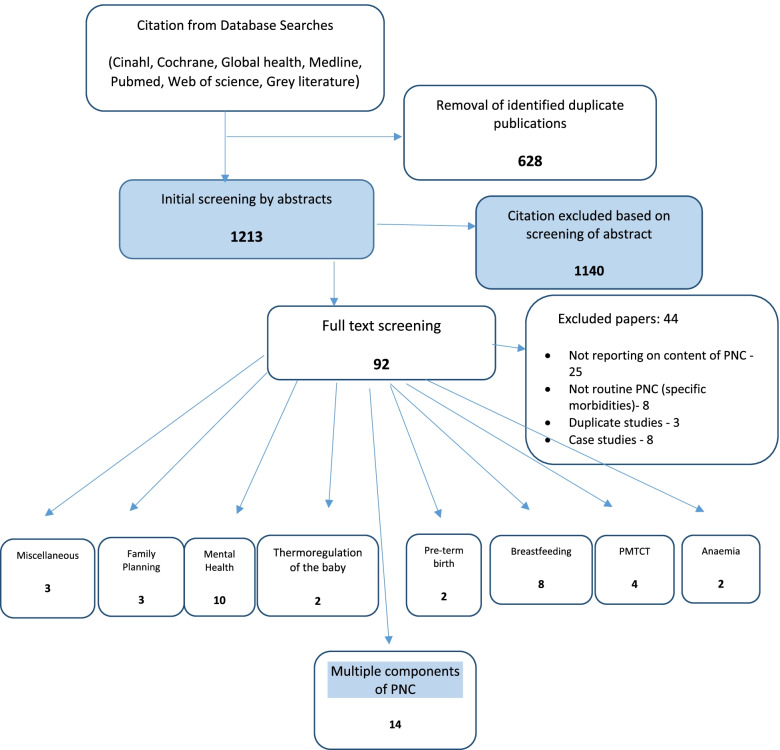
Study selection- PRISMA Flow Diagram (Moher et al., 2009)

Database searches revealed 1213 potentially applicable publications. Duplicates were removed and abstracts reviewed. Exclusion and inclusion criteria was applied and 92 papers were included for full text review. After review 44 papers were excluded and 48 studies included in this review (Fig. 1 – Prisma Diagram). The main exclusion reasons were the policy or study papers were not reporting on content of PNC or were case studies.

Of the 48 included papers 14 reported on multiple components of PNC. This included a systematic review [29], 6 sets of guidelines or policy papers [1, 2, 4, 30–32] and 7 studies or non-systematic reviews [33–39]. (Supplementary Table 2 – Summary of Included studies reporting on multiple components of PNC).

All other papers included (34 papers) reported on only one single component of PNC and were subsequently grouped by 6 themes identified following review including (in order of number of papers included) mental health- postnatal depression (10) [40–49] breastfeeding [8], preventing mother to child transmission of HIV (PMTCT) (4) [50–57], family planning (3) [58–61], care of the pre-term newborn (2) [62–64] and Kangaroo Mother Care (KMC) (2) [67, 68], maternal anaemia (2) [69, 70] and Miscellaneous (3) [71–73] including screening for congenital hip dysplasia, and newborn hip screening and pelvic floor exercises. (Summary Tables available on request).

Quality assessment was performed on the 14 papers (Supplementary table 2), nine were graded as good quality and five assessed to be fair quality. The studies rated fair reported detailed study design methods but not the sampling methods and implications of the study, which compromised their quality. However, all included studies had areas of good quality making them suitable for data extraction.

### Synthesis for papers reporting on single components of PNC

#### Mental health

The largest number of included papers pertained to screening and management of maternal mental health and focused on Postnatal Depression (PND). Several papers report on the effectiveness and/or feasibility of introducing screening for PND. The introduction of screening in India (using the Edinburgh Postnatal Depression Screening- EPDS tool) immediately postpartum and at 6–8 weeks was found to be beneficial in identifying women at risk [40] as did a study in Ethiopia using the WHO self-reporting questionnaire [41]. Similarly, a study in Nepal reported highlighted the need for routine screening for PPD and reported that pregnancy complications and health problems in the baby were risk factors for PND [42].

A systematic review on the effectiveness of psychosocial assessment for the detection and management of PND concluded that assessment helps detect risk factors but those who screen positive and received prevention care for PND did not do better than those who screened positive and received no additional care [43].

A self-care programme consisting of two sessions covering physical and psychosocial wellbeing based on the teach-back method resulted in improved in quality of life during the postnatal period including with regard to improved positive feelings of the mother towards herself and towards her child as well as improved physical health [44]. Women in Australia who had received a short motivational interviewing intervention (including discussion of risk factors for PND, anxiety, low self-esteem) in the postnatal period were four times more likely to seek help for PND in the 12 months after birth [45].

In Iran, weekly support sessions provided by telephone over a period of eight weeks did not result in fewer women screening positive for PND using the EPDS [46] whereas in an RCT lifestyle-based education reduced anxiety and EPDS scores at six weeks postnatally [47]. Educational counselling in additional to routine care (debriefing) for women with adverse birth events did not result in better scores for quality of life, anxiety or depression at six weeks to six months postnatally [48]. Therapist-supported iCBT significantly improves stress, anxiety, and depressive symptoms among postpartum women with small to large effects [49].

#### Breastfeeding

Eight included papers reported on a range of approaches to support breastfeeding. An RCT in the UK examined the effect of skin-to-skin contact care versus none. Although initiation and duration of breastfeeding at four months was not improved, concerns regarding a drop in temp with skin-to-skin care were negated with good maintenance of temperature for the neonate. Both groups got breastfeeding education and support [50]. In a controlled intervention study in Turkey one-to one demonstration using models on how to breastfeed was more effective in preventing cracked nipples than providing an information brochure only [51]. Similarly, in Sweden midwives offered training and support to women at three days, three and nine months postpartum which was reported to result in women enjoying breastfeeding as well as a strengthened maternal relationship with the baby [52]. A one-hour workshop and one-hour counselling session in the first 24 h after birth was reported as effective and beneficial when breast feeding was assessed at four and eight weeks postpartum [53]. In the Gambia women who reported having received counselling, a supportive partner, from a more educated and wealthier background were more likely to intend to practice exclusive breastfeeding [54].

In contrast in another study in the UK support and education counselling after teaching the mother to position the baby herself there was no difference regarding whether the midwife provided further support or was ‘hands-off’ when breastfeeding was assessed at 17 weeks [55]. Evaluation of the effect of peer-to-peer counselling per telephone on breastfeeding duration showed no difference [56]. In an exploratory qualitative study, supporting mother-infant bonding increased the duration of breastfeeding in mothers with babies admitted to a neonatal intensive care unit in Malawi [57].

#### Diagnosis and management of HIV

A study from South Africa [58] highlighted missed opportunities for PMTCT with failure to attend for HIV treatment and FP, lack of TB screening, and women not receiving consistent messages and highlighted the need to address this. In Malawi the need for continued follow -up and care was highlighted through a cohort study with adherence to HIV treatment adequate for 73% or women during pregnancy, 66% in the first 3 months postnatally and 75% during months 4–21 postnatal [59]. The importance of early diagnosis and management of HIV infection in the neonate was demonstrated in Malawi and Thailand [60, 61].

#### Family Planning (FP)

Offering FP as part of immediate PNC was associated with high rates of uptake in a study by *Duncan *et al. among HIV positive women [62]. In a large study from India women who had received a postpartum Intra uterine contraceptive device (IUCD) reported a high level of satisfaction and low level of expulsion (4% at 6 weeks) [63]. In an RCT in the USA contraceptive education by phone, insurance coverage and appointment scheduling did not influence the uptake or not of LARC [64].

#### Preterm and/or LBW babies

A Cochrane review by *Mc Call *et al. looking at thermo-regulation for preterm or low birth weight (LBW) babies reports that using plastic wraps or bags and/or thermo mattresses leads to higher temperature on admission to neonatal units (25 studies included) but that skin-to- skin care remains effective when compared to traditional incubator care [65]. *Sun *et al. recommend the use of a screening algorithm for the prediction of retinopathy of prematurity [66].

#### Prevention of hypothermia

An RCT demonstrated that helping mothers via one-on-one teaching and demonstration of skin-to-skin contact and Kangaroo Mother Care (KMC) improved mother to infant attachment and reduced maternal anxiety [67]. *Nahidi *et al. developed a mother to infant skin to skin contact questionnaire to improve implementation and factors associated [68].

#### Maternal anaemia

The importance of having guidelines for the management of anaemia was reported in a paper from New Zealand with a wide range of approaches noted among midwives especially regarding assessment of iron status [69]. Although two thirds (64.4%) of postnatal women had anaemia in a study from Uganda, the healthcare system had missed the opportunities to effectively address it, such as through the implementation of the policy recommendation for iron and folic acid supplementation [70].

#### Miscellaneous

A systematic review recommends the use of pulse-oximetry screening (POS) to check blood flow in the feet and hands during examination of the newborn baby in the immediate postpartum period for early detection of congenital cardiac heart disease. The reduction in neonatal morbidity and mortality is likely to be more pronounced in low-resource settings where most of these babies are born without a prenatal diagnosis. [71]. A systematic review to examine early dynamic ultrasound (eDUS) screening for hip instability in the first 6 weeks after birth suggests that this could be more effective than clinical examination alone [72]. A systematic review on the effect of pelvic floor muscle exercise reported that this improved sexual desire, arousal, orgasm, and satisfaction in the postpartum period [73].

### Synthesis for papers reporting on multiple components of PNC [14]

*Lassi *et al. included 148 Cochrane and other systematic reviews which identified 61 RMNCH interventions which included eight for routine PNC including: prevention and management of anaemia in the mother, hygienic cord care, prevention of hypothermia with KMC for Low-Birth-Weight babies, newborn immunisation, breastfeeding, family planning, bed nets (ITN) for prevention of malaria, PMTCT for women who are HIV positive. Prevention and management of eclampsia was mentioned as part of ANC only. Home care was mentioned as an approach to delivery of PNC [29].

In a cross-sectional survey among 320 HIV positive postnatal women in Zambia, uptake of infant testing for HIV in the first six weeks was positively associated with maternal uptake of ARVs and, HIV status disclosure to the male partner. Women who reported intimate partner violence (IPV, 40% of the women included in the study) were less likely to have infants tested. Overall, 73% of infants had a test for HIV by 6 weeks. The paper highlights the importance of integration and linking of HIV prevention and management in both the mother and baby and the importance of screening for IPV during PNC [38].

A cluster RCT conducted in Ghana to assess effect of postnatal home visits vs routine PNC available at a healthcare facility and assessed breastfeeding (initiation and exclusive BF), thermoregulation (skin to skin contact, first bath delayed), sleeping under ITN, weighing of the baby and awareness of danger signs to identify the sick baby. Home visits were associated with improved coverage with increased care seeking at the facility in case the baby showed signs of illness (77% in intervention vs 55% in control) [33]*.*

A WHO Technical Working Group reviewed the evidence and reached consensus regarding indicators to assess coverage of key newborn interventions—on two additional indicators for care of the newborn in the immediate postpartum period including regarding; i) thermoregulation recommending drying, delayed bathing, skin-to-skin contact and checking temperature, and ii) cord care—keeping the cord dry versus application of 4% chlorhexidine -with the consensus being the latter needed further research. Additionally, weighing of the baby, breastfeeding and counselling on danger signs in the newborn were agreed as being essential components of PNC [34].

Several studies assessed the availability of quality of PNC. For a comprehensive healthcare facility assessment in Ghana components of (mainly immediate) PNC focused on the newborn and assessed- drying the baby after birth, delaying bathing the baby, prophylactic eye ointment for the baby, initiation of breast feeding, skin to skin contact and KMC for preterm and/or LBW babies [35]. In a before-after study to improve the uptake of intra-partum and postnatal care in Uganda components assessed for PNC included thermo-regulation for the newborn (immediate drying, external warming, skin to skin contact) promotion and provision of hygienic cord care early initiation of breastfeeding and KMC [39].

Two studies looked specifically at care provided in the community or home setting. A study in Iran reported on the effectiveness of community-based PNC which included uptake of PNC in the first week, weight gain during the first 3–7 days, hospitalization rate and management of the sick neonate mainly highlighting the importance of awareness of the danger signs and recognition of the sick baby [37]. Interviews exploring routine practices of home delivery and immediate PNC with women in Ethiopia assessed PNC components including tying the cord immediately after birth, dry cord care, bathing and cleaning the baby birth, and giving the baby water and sugar before initiation of breastfeeding (as non-recommended practice) [36].

### Guidelines

Interventions identified to be essential in the postnatal period for the mother were described in 2011 by the Partnership for Maternal, Newborn and Child Health (PMNCH) and included family planning, prevention and treatment of maternal anaemia, detection and management of postpartum sepsis, PMTCT, immediate thermal care of the baby, initiation of exclusive breastfeeding, hygienic cord and skin care, KMC for preterm and LBW newborns and management of newborns with jaundice. These guidelines also highlighted the level and organisation of care required to provide PNC to women and their newborns [1].

In preparation for the Every Newborn Action Plan (ENAP) 70 indicators were assessed resulting in 10 core and 10 additional indicators being adopted; core indicators were considered those that impact the maternal and/or neonatal morality rate and/or stillbirth rate and include intrapartum skilled birth attendance, early PNC and essential Newborn Care. For PNC treatment of neonatal infections, chlorhexidine for cord care for babies at risk of complications and KMC were identified as essential [31].

The latest WHO Guidelines specifically for PNC [2, 12] address the timing frequency place and content for PNC during the 6 weeks after to birth for mothers and babies and were developed on all available evidence focused on LMICs. Recommended content includes newborn examination, exclusive BF, cord care, delay in bathing, mother and baby staying together, immunisations, examination of the mother (general wellbeing micturition – urinary incontinence, bowel function, perineal care, headache, fatigue, back pain, uterine tenderness and lochia), iron and folic acid supplementation to prevent or manage anaemia in the mother.

Global guidelines for pregnancy, childbirth, postpartum and newborn care include recommendations regarding screening and management of pre-eclampsia and eclampsia; prevention of mother-to-child transmission of HIV; HIV and infant feeding; post-partum depression, and post-partum family planning [12]. The guidelines provide evidence-based recommendations including for the management of endemic diseases like malaria, HIV/AIDS, TB and anaemia. The PNC guidelines recommend administration of Vit K to the newborn and thermal regulation.

The main objective of Salam et al.’s paper was to review the evidence-base for interventions that have a proven positive impact on newborn and maternal health outcomes. In this non-systematic review, for PNC interventions that impacted positively on maternal and neonatal morbidity and mortality included education and provision of family planning, early initiation of and support for exclusive breastfeeding; thermal care or KMC for preterm and/or LBW babies, and hygienic skin and umbilical cord care after birth [30].

In the UK, NICE guidelines [32] outline the care that should be given to women and their babies up to eight weeks after birth. Individual components of clinical care include (but are not limited to) monitoring of blood pressure in the mother, cord care in the baby, administration of Vit K, breastfeeding support. These guidelines also highlight the need to listen to women, be responsive to their needs, taking into consideration the individual needs and preferences of each woman and debriefing after birth.

### Essential Components of PNC and development of signal functions.

From the included papers a total of 22 components of PNC identified as essential were extracted. Results are presented in Table 1 – Components of PNC identified and number of papers supporting each component.

**Table 1:** Components of PNC identified and number of papers supporting each component

**Number**	**Component of PNC**	**Number of papers reporting as essential component**
**Maternal**		
1	Breastfeeding	14
2	Screening and counselling for mental health including depression	13
3	Provide education and advice on ‘danger signs’ in the mother	7
4	Family planning	6
5	Clinical examination of the mother	5
6	Screening for, prevention, and management of Anaemia	4
7	Screening for, prevention, and management of Malaria in the mother	4
8	Screening and management of Gestational Diabetes Mellitus (GDM)	3
9	Screening for pre-eclampsia	2
10	Screening and counselling for domestic violence	2
11	Pelvic floor exercises	1
12	Vitamin A supplementation	1
**Newborn**		
1	Kangaroo Mother Care (KMC)	10
2	Skin-to skin care at birth	10
3	Prevention of maternal to child transmission of HIV (PMTCT)	10
4	Clinical examination of the baby (including congenital abnormalities—screening for hip dysplasia, congenital heart disease)	10
5	Provide education and advice on ‘danger signs’ in the baby	7
6	Screening for, prevention, and management of Malaria in the baby	4
7	Immunisations for the baby and infant	3
8	Vitamin K	3
9	Monitor newborn growth	3
10 **(Total 22)**	Care of the baby born pre-term	3

Peer review and consensus building workshops validated, detailed, and grouped the components which were then developed as developed as proposed signal functions of PNC (Table 2 - Proposed signal functions for PNC with components and outline of required equipment, medication and consumables). Screening for and management of tuberculosis although not identified by the systematic review of the literature was considered a vital and essential component of PNC across all consensus-building workshops along with screening for and management of HIV.

**Table 2:** Proposed signal functions for PNC with essential content and components to be assessed and outline of required equipment drugs and consumables

Signal Function	Components	Essential equipment, drugs and consumables^a^
1.Screening for (pre-)Eclampsia	Measure BP	BP machine and stethoscope
	Test urine for proteinuria	Urine dip-stix, urine containers
2.Prevention and management of Anaemia in the mother	Measure Haemoglobin (Hb)	HemoCue® machine and cuvettes, or laboratory measurement of HB e.g. Coulter counter
	Provide ferrous sulphate with folic acid Nutritional advice	Ferrous sulphate tablets (preferably combined with folic acid)
3.Prevention and management of Malaria in mother and baby	Provide bed nets	Bed nets (preferably insecticide-treated)
	Test for malaria in case of fever and provide treatment for malaria positive women and babies	Thermometer Malaria rapid test, or, malaria microscopy done in lab Drugs for treatment of malaria.^b^ including; artemisinin-based combination therapy (ACT) or artesunate inj or Arthemeter inj for treatment (or as specified in national guidelines)
4.Prevention and management of HIV in mother and baby	Test for HIV	HIV rapid diagnostic test (RDT) (single or combined with syphilis RDT) and/or laboratory
	Provide Prevention of Mother to Child Transmission Care (PMTC) Provide anti-retroviral drugs (ARV) for mother and baby	Drugs^b^ for treatment of HIV positive women and babies pregnancy and prevention of maternal to child transmission (PMTCT)
5.Prevention and management of Tuberculosis (TB) in mother and baby	Test for TB (sputum Ziehl–Neelsen stain or Gen expert point of care testing)	Sputum pots, Lab for sputum Ziehl–Neelsen staining or Gen expert point-of-care testing
	First line TB drugs (preferred)	Drugs^b^ for treatment of tuberculosis (TB) in women and children
	BCG vaccination for newborn	BCG vaccination
6.Screening and counselling for mental health and domestic violence in the mother	Screening tools Counselling services	EPDS, WHO or other self-reporting questionnaire or other tool for mental health screening Screening tool for domestic violence/intimate partner violence Referral pathway for counselling and support
7.Assessment of growth and development of the baby	Assess growth—weight and length	Weighing scale, length measurement table
	Care of the low-birth weight baby	Provide Kangaroo Mother Care (KMC)
	Examination of the new-born baby for congenital abnormalities	Consultation area including for examination and environment facilitating respectful personalised care
8. Cord Care in the baby	Inspection and protection of cord	Chloorhexidine (4%) applied to the cord in areas of high neonatal mortality (≥ 30 per 1000)
9. Vaccination of the baby	Including e.g. Polio, Hepatitis B and BCG at birth and Polio, Rotavirus, Pentavelant at 6 weeks ( subsequent vaccinations at 8, 12 and 16 weeks including Measles Diptheria, Tetanus, Whooping cough)	Vaccins (BCG, Polio-drops, Hepatitis B) BCG and Hepatitis B in areas of high incidence or high risk populations or as per national protocol
10. Clinical examination of the mother	Examination of fundal height and check for lochia Check perineum and any wounds (e.g. Caesarean Section scar) Examination of the legs to check for Deep Vein Thrombosis (DVT)	Examination area with couch b Antibiotics
11. Counselling for and support to exclusive breastfeeding of the baby	Consultation Examination of the breasts	Consultation area including for examination and environment facilitating respectful personalised care
12. Offer contraception/family planning	Consultation	IUD, progesterone only pill, Depo Provera, condoms IUD insertion kit
13. Health promotion and advice for the mother	Advise on perineal care, bladder care, pelvic exercises, resumption of sexual activity	Consultation area including environment facilitating respectful personalised care
14. Debriefing following birth for the mother and counselling for danger signs in mother and baby	Consultation	Consultation area including environment facilitating respectful personalised care

^a^Assumes availability of essential consumables such as non-sterile gloves, needles, syringes or capillary tubes, skin swabs, tourniquet and cotton wool.

^b^All drugs as per national protocol – can vary and needs to be specified for each country.

Throughout the workshops it was highlighted that drug regimens for treatment and prevention of malaria, tuberculosis and HIV should be setting specific and dependent on country practice and policy. Hepatitis B vaccination of the newborn baby is now almost universally recommended but may depend on a country’s national policy and incidence or disease and/or identification of at-risk population. Workshop participants agreed that the proposed PNC signal functions could be used as an important monitoring and evaluation tool including for healthcare facility assessments e.g., to identify the number of healthcare facilities across all levels of care that can provide each of these components and also identify barriers to implementation e.g. lack of human resources, drugs, consumables and equipment The signal functions can also be used as an assessment of service delivery e.g., identification of the proportion of women who received each component during an PNC visit or contact.

## Discussion

### Main findings

As a result of a systematic review of the literature 22 essential components of postnatal care (PNC) were identified of which 12 relate directly to the mother and 10 to the baby. These were synthesised and, following consensus-building with a wide range of stakeholders, were developed into 14 proposed signal functions with the identification of the required equipment, drugs vaccines and consumables to implement each component. As for other care packages in the continuum of care for mothers and children, signal functions of PNC can be used to guide monitoring and evaluation of PNC availability and quality.

We note that in the peer-reviewed literature, guidelines and policy documents the focus has frequently been on components related specifically to neonatal rather than maternal health care. This may be in response to the comparatively high burden of global perinatal and neonatal mortality. However, the health of the baby is directly linked to that of the mother. PNC seeks to address the well-being and health needs of both the mother and her baby during one combined visit or consultation. The proposed signal functions highlight this and do not make a distinction between those that are for the baby and those that are more specifically for the mother. The identified essential components and signal functions include prevention recognition and management of general wellbeing, obstetric complications, medical and infectious diseases that are prevalent as well as social and mental health. Postnatal care is also an important platform to promote exclusive breastfeeding and family planning. It is expected that these essential components of PNC are provided as ‘routine’ for women and babies in the postnatal period to support an optimum recovery for the mother, growth and development of the newborn baby and promote health seeking behaviour for the family. Adaptations can be made where needed depending on the burden of disease in any particular setting and emphasis of focus e.g., for the signal functions pertaining to malaria, TB and HIV. Guidelines for practice will depend on the estimated overall incidence in the population served.

### Strengths and limitations

Postnatal Care is provided as two separate care packages 1) immediate postnatal care at the time of birth and 2) subsequent postnatal care. Postnatal care immediately after birth (in the first few hours) could more logically be considered part of the skilled birth attendance care package or part of intra-partum care and has been described as including prevention of postpartum haemorrhage through active management of the third stage and resuscitation of the newborn if required. Secondly, the care a woman and her baby require at the time of birth and/or immediately after this is very much dependent on the type of birth (vaginal or operative) and whether there are any complications for either the mother or her baby, making it more difficult to define a ‘standard’ or routine care package that would be applicable to all women and babies during the subsequent postnatal period which is commonly defined as the first 42 days after birth.

To the best of our knowledge this is the first systematic review examining specifically what should be considered as the essential content of PNC for the mother and baby to be provided in the first six weeks following birth. The included components are those that are considered part of a comprehensive care package for all women and babies i.e. as part of ‘routine’ PNC. For women and babies with specific complications or underlying morbidity additional PNC components will be required.

### Context in relation to other studies

There is still relatively scant epidemiological information on the specific pregnancy-related burden of disease in the postnatal period. However, this is recognised as a period of risk as well as opportunity for screening, prevention, and management of health problems and to support the wellbeing of the mother and baby. There is emerging evidence that in low-and middle- income settings the burden of morbidity is significant [16, 17]. In high-income settings where the burden of disease is smaller, the emphasis of PNC provision has more recently been on ensuring general well-being of the woman and her baby. This includes and emphasis on social and mental health and debriefing after either complicated or uncomplicated birth, rather than on prevention and management of pregnancy complications or infectious diseases.

For the purpose of developing globally relevant signal functions and, based on the results of our systematic review of the literature, the signal functions proposed in this study seek to be comprehensive and recognise the need to address three major infectious diseases (HIV, tuberculosis and malaria) as well as obstetric conditions, medical conditions, mental and social health. For those populations with a low prevalence of HIV, tuberculosis and/or malaria country adaptations can be made. It would be helpful to have agreed international cut-off points of prevalence above which screening for, and management of certain infectious diseases should be included as essential components of PNC. A useful comparison is the recommendations regarding whether or not to screen for tuberculosis as part of antenatal care which is guided by estimated country level prevalence of tuberculosis [8]. Screening for HIV is almost universally recommended as part of ANC and may not have to be repeated as part of PNC in countries with a low prevalence.

Examination of the baby to check for any congenital abnormalities and/or illness is an important part of PNC. In many settings an anomaly ultrasound scan is routinely offered as part of ANC. Whether or not this is provided, it remains important to ensure a full body examination of the baby as part of PNC. In high income settings additionally laboratory testing is carried out (e.g. a Guthrie or ‘heel prick’ test to check for phenylketonuria) and a routine hearing test is carried out on all newborn babies to identify deafness.

Discussion regarding the need for and/or effectiveness of de-briefing after traumatic birth as well as general de-briefing and information sharing after uncomplicated birth have informed the development of the relevant proposed signal function and was recognised by stakeholders during consultation to be a new and emerging component of PNC which requires further attention [74, 75].

We conducted an earlier and separate systematic review and consensus-building to identify the essential components of ANC with the development of 15 proposed signal functions [26]. We note that there is an overlap in content and therefore the signal functions developed for both ANC and PNC and we recommend these are combined for the purposes of monitoring and evaluation as well as training of healthcare providers. In most setting ANC and PNC are provided in similar settings and by the same cadres of healthcare providers including for the main part community- and/or facility-based nurse-midwives.

### Implications for policy and practice

It is recognised that PNC is being delivered by a wide range of healthcare providers, including those at facility- and community-level. For a full content of effective PNC to be delivered these healthcare providers need to have the necessary equipment, dugs, consumables as well as up-to-date knowledge and skills in all aspects of PNC. As it can be expected that many women are home-bound especially in the early postnatal period, there is a need to provide care at home or very close to home and models for this may require further development [76]. Recently there have been suggestions that community-based healthcare workers and/or volunteers may be better placed to provide PNC. However, an initial mapping shows that few of such cadres are adequately trained for, competent in, legislated and supported to provide all of the essential components of either ANC or PNC [77]. Other forms of community support are effective such as mother to mother support for continuation of exclusive breastfeeding, practical and emotional support from partners, family and the wider community to enhance wellbeing and promote a positive experience of the postnatal period [78, 79].

The importance of screening for social (including gender-based violence) as well as mental health problems (including depression) during the antenatal as well as postnatal period is recognised globally [17, 80]. A variety of screening tools is available currently to assess wellbeing and mental health and it will be important to establish which is most effective and feasible to use in each specific setting. In some cases, translation into a local language and/or socio-cultural adaptation of tools is still needed. However, our systematic review highlighted several studies from countries where such screening has now been successfully introduced. Screening for GBV and/or intimate partner violence (IPV) is problematic in many settings and not accepted practice either for the healthcare provider or the woman attending for care [81, 82]. WHO guidelines for ANC recommend screening for GBV/IPV in settings where women can receive care and a referral pathway is established [8]. However, although recommended practice, we note that the current WHO guidelines for PNC make no specific recommendation regarding GBV [12].

### Future research and unanswered questions

PNC coverage is defined as the number of women aged 15–49 years with a live birth who have postnatal contact with a health-care provider within two days of birth as a proportion of the total number of women aged 15–49 with a live birth. This information is collected from Demographic Health Surveys (DHS), Reproductive Health Surveys (RHS), Multiple Indicator Cluster Surveys (MICS), or other types of household surveys that collect data using nationally representative population samples and standardised questionnaires. Population-based household surveys are the preferred data source in settings that have a low utilization of healthcare facility services. However, such surveys are generally expensive and may be difficult to conduct.

The availability and uptake of essential health services coverage (SDG indicator 3.8.1) is an important measurement [83]. Thus, the Universal Health Coverage (UHC) index includes 16 essential health services as indicators of the equity and level of UHC. For reproductive, maternal, neonatal and child health (RMNCH) these are: family planning, antenatal care, delivery care, full child immunisation, and care for pneumonia in children [83]. We note that unlike ANC coverage, PNC coverage is not reported as an indicator in the annual World Health Statistics reports.

The available indicators for PNC are obtained for populations through a variety of different data sources, the most important of which are household surveys. Regarding PNC coverage, in both the MICS and DHS what is measured is; 1) the proportion of women who recall having received PNC within 2 days of giving birth and 2) who (which type of healthcare provider) provided PNC. Since 2013, in DHS (phase 7) what is asked is: did the woman receive PNC in the 2 days after birth, who provided this and where. For content what is included is: if the healthcare provider examined the cord, measured the baby’s temp, counselled on danger signs and observed breastfeeding [84]*.*

Given the importance of PNC as a key healthcare package for the prevention and management of morbidity and mortality in women of reproductive age and in the newborn, it will be important to reach global consensus on more effective routine monitoring of PNC coverage and content. The signal functions developed could be used: 1) for health facility assessment; 2) to identify health system barriers to implementation; 3) as a component of the service delivery assessment and, 4) for assessment of quality of care. Further research regarding the acceptability and feasibility of the application of the signal functions proposed in this study for the effective monitoring and evaluation of availability and quality of PNC is needed.

## Conclusion

Globally the proportion of mothers and babies who receive PNC is significantly lower than those who receive ANC and this constitutes a missed opportunity. Reasons for this could be that PNC is not accessible, not available and/or not of good quality. The focus has to date been mainly on the number and timing of PNC visits rather than what is provided to the mother and baby during these visits or contacts. More attention should be given to content if PNC is to have the required impact on maternal and neonatal morbidity and mortality. We propose a set of signal functions that could be used to monitor and evaluate content of PNC. There is ample evidence for the urgent need to address aspects of care that are disrespectful and of poor quality and that are likely to contribute significantly to the current low uptake of postnatal care globally. Similarly, without the required essential content for the mother and her baby quality of care cannot be provided.

## Supplementary Information

**Additional file 1:** **Table S1.** Search Strategy.

**Additional file 2:** **Supplementary Table 2.** Summary of included studies reporting on multiple components of Postnatal Care (PNC).

## Data Availability

NA

## Abbreviations

ANC: Antenatal Care
GBV: Gender-based violence
IPV: Intimate Partner Violence
PNC: Postnatal Care
PND: Postnatal Depression
UHC: Universal Health Coverage
RCT: Randomised controlled trial
