# Supplementary material for: Essential components of postnatal care – a systematic literature review and development of signal functions to guide monitoring and evaluation
Source: BMC Pregnancy Childbirth. 2022 May 28;22:448. doi: 10.1186/s12884-022-04752-6 (PMC9148465; doi:10.1186/s12884-022-04752-6)
Supplement: Supplementary file 1 — Additional file 1: Table S1. Search Strategy. [file 12884_2022_4752_MOESM1_ESM.docx]

**Table S1: Search Strategy**

| **Data bases:** Cinahl, Cochrane, Global Health, Medline, PubMed and Web of Science. |
| --- |
| **Search terms**  (“postnatal”) or (“postpartum”) or (“PNC”) or (“after pregnancy”)  **and**  (“essential components”) or (“essential interventions”) or (“signal functions”) or (“key indicators”) or (“clinical indicators”) or (“routine care”) |
| **Headings added depending on individual databases**  **Cinahl-** Subheadings: (“clinical indicators”) and (“postnatal”) or (postnatal care”)  **Cochrane**- MESH: (“standard of care”) or (“care bundles”)  **Global health**- Thesaurus: (“DE indicators”) or (“DE clinical components”) and (“DE postpartum”) or (“DE postnatal period”)  **Medline**-Mesh headings: ( quality indicators”) and (“postnatal”) |
