# Supplementary material for: Essential components of postnatal care – a systematic literature review and development of signal functions to guide monitoring and evaluation
Source: BMC Pregnancy Childbirth. 2022 May 28;22:448. doi: 10.1186/s12884-022-04752-6 (PMC9148465; doi:10.1186/s12884-022-04752-6)
Supplement: Supplementary file 2 — Additional file 2: Supplementary Table 2. Summary of included studies reporting on multiple components of Postnatal Care (PNC). [file 12884_2022_4752_MOESM2_ESM.docx]

**Supplementary Table 2** : **Summary of included studies reporting on multiple components of Postnatal Care (PNC)**

| **Title** | **Authors and Year Published** | **Type of study/paper** | **Research Methods** | **Countries (HIC/LMIC)** | **No of women/healthcare providers/ population included** | **Key Findings** | **Quality assessment score/grading** |
| --- | --- | --- | --- | --- | --- | --- | --- |
| **Systematic reviews** | | | | | | |  |
| 1. Interventions to Improve Neonatal Health and Later Survival: An Overview of Systematic Reviews | Lassi Z, Middleton PF, Crowther C, Bhutta ZA (2015) | Review of Systematic reviews | 148 Cochrane and other systematic reviews | LMICs and HICs | 61 reproductive, maternal, newborn and child health interventions | This SR identifies 4 PNC interventions and 12 newborn interventions of which 8 are routine PNC | 32  Good |
| **Studies and non-Systematic Reviews** | | | | | | |  |
| 1. Effect of the Newhints home-visits intervention on neonatal mortality rate and care practices in Ghana: a cluster randomised controlled trial | Kirkwood B, Manu A, Asbroek A, Soremekun S, Weobong B, Gyan T, Danso, S Amenga-Etego S, Tawiah-Agyemang C, Owusu-Agyei S, Hill Z.  (2013) | Evaluation of a home-visits strategy in sub-Saharan Africa by assessing the effect on all-cause neonatal mortality rate (NMR) and essential newborn-care practices. |  | Ghana | The Newhints cluster randomised trial was undertaken in 98 zones in seven districts in the Brong Ahafo Region, Ghana. 49 zones were randomly assigned to the Newhints intervention and 49 to the control intervention by use of restricted randomisation with stratification to ensure comparability between interventions. | The largest increase was for care-seeking bednet use during pregnancy (money saved for delivery or emergency transport arranged in advance for facility birth assistant for home delivery washed hands with, initiation of breastfeeding in less than 1 h of birth skin to skin contact, first bath delayed for longer than 6 h, exclusive breastfeeding for 26-32 days (and baby sleeping under bednet for 8-56 days. | 32  Good |
| 1. Measuring coverage in MNCH: indicators for global tracking of newborn care | Moran AC, Kerber K, Sitrin D, Guenther T, Morrissey CS, Newby H, Fishel J, Yoder PS, Hill Z, Lawn JE.  (2013) | Newborn Indicators Technical Working Group (TWG) developed consistency in measurement of postnatal care for women and newborns and of immediate care behaviours and practices for newborns. | Qualitative research and cross-sectional household surveys with pre/post design using community cluster sampling were conducted | Ghana, Nepal, Bangladesh and Malawi | In Ghana (40 birth narratives, four focus groups, ten health worker interviews. In Bangladesh and Malawi (84 and 80 narratives/questionnaires, respectively).1,500 women for survey (600 Nepal, 800 India) |  | 33  Good |
| 1. Quality along the continuum: a health facility assessment of intrapartum and postnatal care in Ghana | Nesbitt RC, Lohela TJ, Manu A, Vesel L, Okyere E, Edmond K, Owusu-Agyei S, Kirkwood BR, Gabrysch S.  (2013) | To evaluate quality of routine and emergency intrapartum and postnatal care using a health facility assessment, and to estimate “effective coverage” of skilled attendance in Brong Ahafo, Ghana. | Conducted an assessment of all 86 health facilities in seven districts in Brong Ahafo. | Ghana | 86 healthcare facilities | Using performance of key signal functions and the availability of relevant drugs, equipment and trained health professionals, we created composite quality categories in four dimensions: routine delivery care, emergency obstetric care (EmOC), emergency newborn care (EmNC) and non-medical quality. | 30  Fair |
| 1. A qualitative study exploring newborn care behaviours after home births in rural Ethiopia: implications for adoption of essential interventions for saving newborn lives | Salasibew, Mihretab M, Filteau S, Marchant T. (2014) | in this study, we explored the sequence of immediate newborn care practices and associated beliefs following home deliveries in rural communities in Ethiopia. | Qualitative research using semi-structured interviews and focus group discussions | Ethiopia. | 26 semi-structured interviews and 2 focus group discussions |  | 27  Fair |
| 1. Effects of community-based newborn care intervention on neonate health status in a district of Tehran (Iran) | Nayeri F, Dalili H, Shahzadeh Fazeli K, Delbarpoor Ahmadi S, Akrami F, Esmailnia T, Habibelahi A, Shariat M.  (2016) | A community and healthcare centre-based study was carried out from January 2011 through September 2014. | Demographic data of mothers and infants were recorded in questionnaires before and after intervention. | Tehran Iran | The populations in the before and after intervention groups were 274 and 250, respectively. | Interventions were implemented in hospitals, participants' homes, and health centres. The primary outcomes were comparison of mean birth weight, weight gain during the first 3-7 days, first week visit rate, hospitalization rate between the before and after intervention groups. | 27  Fair |
| 1. Improving the quality of maternity services in Uganda through accelerated implementation of essential interventions by healthcare professional associations | Spira C, Kwizera A, Jacob S, Amongin D, Ngonzi J, Namisi CP, Byaruhanga R, Rushwan H, Cooper P, Day-Stirk F, Berrueta M, García-Elorrio E, Belizán JM  (2017) | To assess whether the implementation of a package of activities through the joint action of the three international healthcare professionals associations (HCPAs) increased the use of intrapartum and postnatal Essential interventions (EIs) in two hospitals in Uganda. | A non-controlled before-and-after study was undertaken to evaluate the effect of a package of activities designed to change practice relating to nine EIs among providers. Coverage of the EIs was measured in a 3-month pre-implementation period and a 3-month post-implementation period in 2014. | Uganda | Overall, 4816 women were included. |  | 32  Good |
| 1. Barriers to uptake of early infant HIV testing in Zambia: the role of intimate partner violence and HIV status disclosure within couples. | Hampanda KM, Nimz AM, Abuogi LL. (2017) | The aim of this study was to explore how gender power dynamics within couples affect HIV-positive women’s uptake of early infant HIV testing. | Cross-sectional survey. | Zambia | 320 HIV-positive married postpartum women | Domestic relationship dynamics, including emotional violence and HIV status disclosure to the male partner, may play an important role in maternal uptake of early infant HIV testing. | 29  Fair |
| **Guidelines and Policy Papers** | | | | | | |  |
| 1. Essential Interventions, Commodities and Guidelines for Reproductive, Maternal, Newborn and Child Health | Partnership for Maternal Newborn and Child Health (PMNCH) with World Health Organization (WHO) and Aga Khan University (2011) | Policy document | A total of 142 RMNCH interventions were identified, assessed, and selected for this review | LMIC |  | Consensus was reached on the content of RMNCH packages of interventions at each level of the health system across the continuum of care including PNC. | 33  Good |
| 1. WHO Recommendations on Postnatal Care of the Mother and Newborn. | Geneva: World Health Organization; (2013) | Guidelines | Evidence review and consensus building document | LMIC |  | WHO guidelines on postnatal care based on all available evidence. The guidelines focus on postnatal care of mothers and newborns in resource-limited settings in low- and middle-income countries. | 33  Good |
| 1. Essential childbirth and postnatal interventions for improved maternal and neonatal health | Salam RA, Mansoor T, Mallick D, Lassi ZS, Das JK, Bhutta ZA.  (2014) | The main objective of this paper is to review the evidence-based childbirth and post-natal interventions which have a beneficial impact on maternal and newborn outcomes |  | LMIC |  | A compilation of existing, new and updated interventions designed to help physicians and policy makers and enable them to reduce the burden of maternal and neonatal morbidities and mortalities. | 29  Fair |
| 1. Count every newborn; a measurement improvement roadmap for coverage data | Moxon SG, Ruysen H, Kerber KJ, Amouzou A, Fournier S, Grove J, Moran AC, Vaz LM  (2015) | Policy paper | In a multistage process, a matrix of 70 indicators were assessed by the *Every Newborn* steering group. Indicators were graded based on their availability and importance to ENAP, resulting in 10 core and 10 additional indicators | LMIC |  | The roadmap presents a unique opportunity to strengthen routine health information systems, crosslinking these data with civil registration and vital statistics and population-based surveys. | 32  Good |
| 1. Pregnancy, childbirth, postpartum and newborn care: a guide for essential practice. Pregnancy, childbirth, postpartum and newborn care: a guide for essential practice | WHO  (2015) | Guidelines | Evidence review and consensus building document | LMIC |  | The aim of this guideline is to provide evidence-based recommendations to guide health care professionals in the management of women during pregnancy, childbirth and postpartum, and newborns, and post abortion, including management of endemic diseases like malaria, HIV/AIDS, TB and anaemia. | 33  Good |
| 1. Postnatal care | NICE guidelines (2020) | PNC Guideline | Evidence based care review | HIC |  | This guideline covers the routine postnatal care that women and their babies should receive in the first 6 weeks after the birth. It includes the organisation and delivery of postnatal care, identifying and managing common and serious health problems in women and their babies, how to help parents form strong relationships with their babies, and baby feeding. | 35  Good |
